# Genome-wide identification, phylogenetic and expression pattern analysis of Dof transcription factors in blueberry (*Vaccinium corymbosum* L.)

**DOI:** 10.7717/peerj.14087

**Published:** 2022-10-03

**Authors:** Tianjie Li, Xiaoyu Wang, Dinakaran Elango, Weihua Zhang, Min Li, Fan Zhang, Qi Pan, Ying Wu

**Affiliations:** 1 Tianjin Agricultural University, Tianjin, China; 2 Inner Mongolia Minzu University, Mongolia, China; 3 Iowa State University, Ames, United States of America

**Keywords:** Dof transcription factor, Phylogeny, Expression pattern analysis, Blueberry

## Abstract

**Background:**

DNA binding with one finger (Dof) proteins are plant-specific transcription factor (TF) that plays a significant role in various biological processes such as plant growth and development, hormone regulation, and resistance to abiotic stress. The Dof genes have been identified and reported in multiple plants, but so far, the whole genome identification and analysis of Dof transcription factors in blueberry (*Vaccinium corymbosum* L.) have not been reported yet.

**Methods:**

Using the Vaccinium genome, we have identified 51 *VcDof* genes in blueberry. We have further analyzed their physicochemical properties, phylogenetic relationships, gene structure, collinear analysis, selective evolutionary pressure, cis-acting promoter elements, and tissue and abiotic stress expression patterns.

**Results:**

Fifty-one *VcDof* genes were divided into eight subfamilies, and the genes in each subfamily contained similar gene structure and motif ordering. A total of 24 pairs of colinear genes were screened; *VcDof* genes expanded mainly due to whole-genome duplication, which was subjected to strong purifying selection pressure during the evolution. The promoter of *VcDof* genes contains three types of cis-acting elements for plant growth and development, phytohormone and stress defense responsiveness. Expression profiles of *VcDof* genes in different tissues and fruit developmental stages of blueberry indicated that *VcDof2* and *VcDof45* might play a specific role in anthesis and fruit growth and development. Expression profiles of *VcDof* genes in different stress indicated that *VcDof1*, *VcDof11*, and *VcDof15* were highly sensitive to abiotic stress. This study provides a theoretical basis for further clarifying the biological function of *Dof* genes in blueberry.

## Introduction

Blueberry is a perennial woody plant that belongs to the genus Vaccinium. It is one of the five healthy fruits recommended by the Food and Agriculture Organization of the United Nations; it is rich in anthocyanins, vitamins, flavonoids, and other active ingredients (Kalt et al., 2020; Li et al., 2020). It has the functions of protecting eyesight, preventing type 2 diabetes, anti-aging, and anti-cancer (Ma et al., 2018; Ono-Moore et al., 2016; Istek & Gurbuz, 2017). Due to various health benefits of blueberries and awareness of healthy diets, consumption of blueberries and their derivatives increased tremendously. So far, blueberry has grown in 71 countries (Gallardo et al., 2018). Rapidly changing climatic conditions may drastically affect the global production of blueberries in the coming years. So, understanding and identifying genetic factors’ response to biotic and abiotic stress tolerance is imperative. Research shows that salt and drought stress alone in blueberry causes 25–30% yield losses (Wang et al., 2021a; Wang et al., 2021b).

A transcription factor (TF) is a DNA binding protein that can specifically interact with cis-acting elements of genes and regulate the specific expression of target genes (Zhang & Hou, 2021). Understanding its structure and function is very important to recognize the biological role of the gene in the given context (Chen et al., 2017). DNA binding with one finger (Dof) TFs consist of 200–400 amino acids (Gupta et al., 2015). Dof includes the DNA-binding domain and domain involved in transcriptional activation or repression and exhibits regulatory roles in nuclei (Chattha et al., 2020; Liu et al., 2020). But, except for the highly conserved domain of Dof, the amino acid sequences are not well conserved, this creates a greater diversity across family members, and individual proteins exhibit very divergent sequences (Wu et al., 2016; Cominelli et al., 2011; Yang et al., 2011), which may be an important reason for the functional diversity of Dof proteins and involved in varied plant physiological and biochemical processes (Yanagisawa, 2002).

In 1993, the first Dof transcription factor *ZmDof1* was identified and cloned in maize (Yanagisawa & Izui, 1993). With the recent developments in genome sequencing and bioinformatics capabilities, multiple Dof TFs have been identified in *Arabidopsis thaliana*, *Solanum tuberosum*, *Solanum lycopersicum*, *Oryza sativa*, and *Zea mays* (Lijavetzky, Carbonero & Vicente-Carbajosa, 2003; Venkatesh & Park, 2015; Cai et al., 2013; Jiang et al., 2012; Moreno-Risueno et al., 2007). Dof TFs have been shown to play an important role in plant growth and development, primary and secondary metabolism, hormone regulation, and abiotic stress resistance (Renau-Morata et al., 2017; Yanagisawa, 2004; Skirycz et al., 2007; Li et al., 2022). Specifically, inducible overexpression of *AtDof5.4/OBP4* in Arabidopsis promoted early endocycle onset, inhibited cell expansion, and reduced cell size and number, resulting in dwarf plants (Xu et al., 2016). *AtDof4.7* regulates the expression of cell wall hydrolysis enzymes; overexpression of *AtDof4.7* causes an abscission-related polygalacturonase gene *PGAZAT* down-regulation, which affects the shedding of flower organs (Wei et al., 2010). *AtDof6* can regulate seed germination by interacting with TCP14 protein and affecting ABA anabolism (Rueda-Romero et al., 2012). Overexpression of *GmDof4* and *GmDof11* genes in *Glycine max* increased the content of total fatty acids and lipids of transgenic Arabidopsis seeds (Wang et al., 2007). Overexpression of tomato Dof gene family member *TDDF1* in plants can improve tomato tolerance to drought, salt, and various hormone treatments (Ewas et al., 2017). In *Tamarix hispida*, *ThDof1.4* enhances proline levels, reactive oxygen species (ROS) scavenging capacity, and tolerance to salt and osmotic stress (Zang et al., 2017). Many studies have shown that the Dof transcription factor gene family plays an important role in plant growth, development, and resistance to abiotic stress.

The haplotype-phased genome assembly of blueberry was published in 2019 (Colle et al., 2019). So far, there are few reports on the role of Dof TFs in abiotic stress tolerance. Therefore, we aim to identify Dof TFs in blueberry using the whole genome of blueberry, and we further analyzed gene structure, phylogenetic relationships, collinear analysis, and the expression pattern of *VcDof* genes under abiotic stress in different tissue types was analyzed using RNA-Seq and qRT-PCR. In summary, the present research provides important information on the potential function of the blueberry Dof TFs in abiotic stress tolerance.

## Materials & Methods

### Identification and characterization of Dof transcription factors in blueberry

The blueberry genome and annotation data were downloaded using the Vaccinium database (GDV, https://www.vaccinium.org). The Dof TFs protein sequences of Arabidopsis and rice were used as query sequences (https://phytozome-next.jgi.doe.gov/). The blueberry genome was compared by Basic Local Alignment Search Tool Protein (BLASTP) in a Linux system, and the screening threshold *E*-value was 1e−5. The protein sequences of candidate genes were obtained. Furthermore, the Dof domain, PF02701, was used to search the blueberry genome using HMMER software (Potter et al., 2018). By integrating the results of the above two steps, the sequences of these genes were submitted to SMART (http://smart.embl.de/smart/batch.pl) and NCBI-CDD (https://www.ncbi.nlm.nih.gov/cdd/) to remove redundant and non-conservative genes (Letunic & Bork, 2018; Lu et al., 2020). Then, the gene sequences of blueberry Dof TFs were obtained, named according to their position on the chromosome scaffold.

The amino acid number, theoretical isoelectric point (pI), and molecular weight (MW) of *VcDof* genes were analyzed online using the ProtParam tool (https://web.expasy.org/protparam). Subcellular localization information of *VcDof* genes was predicted using CELLO (http://cello.life.nctu.edu.tw) and WoLF PSORT (https://wolfpsort.hgc.jp).

### Multiple sequence alignments and phylogenetic analysis

The protein sequences of Dof TFs in *Arabidopsis thaliana* and *Oryza sativa* were downloaded from NCBI (https://www.ncbi.nlm.nih.gov/) and phytozome (https://phytozome-next.jgi.doe.gov/). Multiple sequence alignments (MSA) were performed using MegaX software (Kumar et al., 2018). Based on multiple sequence alignment results, a phylogenetic tree was constructed using the maximum likelihood (ML) method; the optimal fitting model was selected as JTT+G, and the number of Bootstrap tests was adjusted to 1000. The resulting phylogenetic tree file was enhanced using iTol (https://itol.embl.de/, Letunic & Bork, 2021).

### Gene structure analysis of the Dof transcription factors

Based on the blueberry genome structure annotation information file, the number and location of exons/introns of *VcDof* genes were counted by Gene Structure Display Server (GSDS2.0, http://gsds.gao-lab.org, Hu et al., 2015a; Hu et al., 2015b). The conservative motif of blueberry Dof protein was analyzed online by Maximization for Motif Elicitation program (MEME, https://meme-suite.org/meme/tools/meme, Bailey et al., 2015). The TBtools were used for visualization (Chen et al., 2020).

### Collinearity analysis of Dof transcription factors in blueberry

Duplication events of *VcDof* genes were analyzed by the MCscanX (Tang et al., 2008) and visualized in TBtools. Using Ka/Ks ratio to display evolutionary selection pressure between collinearity gene pairs, Ka/Ks>1, = 1, <1 indicated positive selection, neutral evolution, and purifying selection, respectively. Further, T = Ks/2 *λ* (T: calculates divergence time; Mya: million years; *λ*: replacement rate, *λ* = 1.3 × 10^−8^) was used to compute the approximate date of duplication and divergence events (Colle et al., 2019).

### Promoter cis-acting element analysis

The 2,000 bp promoter region upstream of *VcDof* genes were obtained from the blueberry genome, and the cis-acting elements in the promoter region were analyzed using the tool PlantCARE (http://bioinformatics.psb.ugent.be/webtools/plantcare/html).

### Expression profile of Dof transcription factors in different tissues

Using the RNA-Seq data, the expression level of *VcDof* genes in different tissues and fruit development stages were identified (https://www.vaccinium.org/; NCBI accession number: PRJNA494180). The transcript abundance was estimated using FPKM (fragments per kilobase per million measure) after log_2_ conversion (Colle et al., 2019). Hierarchical clustered heatmaps and visualization were done using the TBtools.

### Plant material and experimental treatments

In this study, the saplings of the northern highbush blueberry ‘Bluecrop’ preserved in the laboratory were used as experimental materials, and the pots were filled with nutrient soil and vermiculite (volume ratio 1:1). The effective components of nutrient soil were nitrogen 140 mg/kg^−1^, phosphorus 100 mg/kg^−1^, potassium 180 mg/kg^−1^, organic matter content 91.3%, PH 5.5. The biennial sapling plants were placed in the greenhouse of Tianjin Agricultural University. The deionized water and 1/2 Hoagland nutrient solution were regularly irrigated to make the plants grow under the optimal growth conditions.

This experimental design was a completely randomized design with three treatments. The specific treatments were as follows: 150 mMol/L NaCl was used to irrigate blueberry plants to simulate salt stress; 20% PEG6000 was used to irrigate the plants to simulate drought stress; and ABA hormone 100 µMol/L was used to spray blueberry leaves to simulate adversity stress. After 0(control), 3, 6, 12, and 24 h of each treatment, leaf samples were collected. Triplicate leaf samples were collected for each time point and treatment and frozen in liquid nitrogen. The samples were stored in an ultra-low temperature freezer at −80 °C (Bano et al., 2021; Han, Wu & Li, 2021).

### RNA isolation, cDNA preparation, and quantitative RT-PCR analysis

The RNA extraction was done using the Easypure Plant RNA Kit (TransGen Biotech, China). After gel electrophoresis detected clear RNA bands and apparent separation, cDNA was synthesized using the kit PrimeScript TMRT Master Mix (TaKaRa BIO INC., Japan). The blueberry EIF (*VcEIF*) gene was used as the reference (Deng, Li & Sun, 2020), and the specific primers for the target gene and the reference gene were designed using Premier 5.0 software. The instrument used for qRT-PCR was qTOWER 2.2 (Analytik Jena, Germany), and the qRT-PCR reaction was performed using the iTaq Universal SYBR^®^ Green Supermix (Bio-Rad INC., USA). The relative expression levels were calculated as 2 ^−ΔΔCt^ (Livak & Schmittgen, 2001), the significance of the difference between each treatment group compared to control was determined using one-way ANOVA in SPSS 26.0 (SPSS Inc., USA), error bars indicate standard deviation, and asterisks indicate significant differences between the treatments and control, ^∗^*p* ≤ 0.05, ^∗∗^*p* ≤ 0.01, ^∗∗∗^*p* ≤ 0.001.

## Results

### Genome-wide identification of Dof transcription factors in blueberry

The 51 blueberry Dof TFs were identified and named *VcDof1* to *VcDof51* according to the position of the genes on the chromosome scaffold (Table 1). The length of amino acids varied greatly, with VcDof40 the longest, containing 493 amino acids, and VcDof36 the shortest, containing 118 amino acids. The analysis of physicochemical properties using ProtParam showed that the molecular weight of *VcDof* genes was between 13,621.8 and 53,701.66 Da. The theoretical isoelectric point was 4.56–10.56 for both acidic and alkaline proteins. The theoretical isoelectric point of *VcDof15* is the smallest, showing a higher precipitation coefficient. The theoretical isoelectric point of *VcDof36* is the largest, suggesting that it has strong solubility and weak precipitation ability. Subcellular localization prediction showed that most *VcDof* genes were located in the nucleus.

**Table 1:** The basic information of Dof TFs in blueberry.

**Gene name**	**Gene ID**	**Chromosome location**	**CDS (bp)**	**Length (aa)**	**pI**	**Molecular weight (Da)**	**Subcellular location**
*VcDof1*	VaccDscaff3-snap-gene-159.14	VaccDscaff3:15954245-15956961	1200	399	8.31	44,373.94	Nuclear
*VcDof2*	VaccDscaff4-augustus-gene-345.25	VaccDscaff4:34550915-34552763	876	291	8.1	31,800.02	Nuclear
*VcDof3*	VaccDscaff8-augustus-gene-261.30	VaccDscaff8:26158034-26160021	918	305	9.26	33,049.91	Nuclear
*VcDof4*	VaccDscaff9-augustus-gene-161.15	VaccDscaff9:16127607-16130475	801	266	9.18	29,743.08	Nuclear
*VcDof5*	VaccDscaff10-snap-gene-247.18	VaccDscaff10:24688121-24689836	906	301	9.26	32,614.39	Nuclear
*VcDof6*	VaccDscaff11-processed-gene-136.2	VaccDscaff11:13655066-13655560	495	164	9.43	18,177.54	Nuclear
*VcDof7*	VaccDscaff11-snap-gene-158.12	VaccDscaff11:15805973-15808315	1071	356	8.96	39,331.62	Nuclear
*VcDof8*	VaccDscaff11-processed-gene-168.11	VaccDscaff11:16838900-16843433	612	203	9.85	22,095.71	Nuclear
*VcDof9*	VaccDscaff11-processed-gene-307.12	VaccDscaff11:30754783-30756949	951	316	7.63	34,553.12	Nuclear
*VcDof10*	VaccDscaff11-augustus-gene-330.45	VaccDscaff11:33025388-33027124	867	288	9.43	30,770.94	Nuclear
*VcDof11*	VaccDscaff12-processed-gene-81.3	VaccDscaff12:8170002-8170682	681	226	6.37	24,042.46	Nuclear
*VcDof12*	VaccDscaff13-augustus-gene-79.30	VaccDscaff13:7890522-7894462	1470	489	5.74	53,351.37	Nuclear
*VcDof13*	VaccDscaff13-augustus-gene-135.30	VaccDscaff13:13508244-13511349	957	318	9.04	34,095.62	Nuclear
*VcDof14*	VaccDscaff13-augustus-gene-254.20	VaccDscaff13:25461888-25465267	1407	468	5.57	51,450.94	Nuclear
*VcDof15*	VaccDscaff14-processed-gene-266.8	VaccDscaff14:26675412-26676173	762	253	4.56	28,167.22	Nuclear
*VcDof16*	VaccDscaff15-snap-gene-139.15	VaccDscaff15:13899895-13902343	1062	353	9.07	39,109.51	Nuclear
*VcDof17*	VaccDscaff15-processed-gene-145.13	VaccDscaff15:14528524-14530344	795	264	9.3	28,571.85	Nuclear
*VcDof18*	VaccDscaff15-augustus-gene-292.25	VaccDscaff15:29225641-29227947	954	317	7.21	34,696.27	Nuclear
*VcDof19*	VaccDscaff15-processed-gene-320.11	VaccDscaff15:32030644-32032478	1281	426	9.88	46,664.59	Nuclear
*VcDof20*	VaccDscaff16-augustus-gene-64.37	VaccDscaff16:6408947-6412982	1395	464	5.93	50,683.97	Nuclear
*VcDof21*	VaccDscaff17-augustus-gene-280.24	VaccDscaff17:28047716-28050042	963	320	6.52	35,155.14	Nuclear
*VcDof22*	VaccDscaff17-processed-gene-380.10	VaccDscaff17:38033194-38035650	885	294	8.11	32,152.67	Nuclear
*VcDof23*	VaccDscaff19-augustus-gene-7.37	VaccDscaff19:722925-724763	873	290	8.81	32,131.57	Nuclear
*VcDof24*	VaccDscaff19-processed-gene-223.3	VaccDscaff19:22351441-22352415	975	324	9.20	35,667.63	Nuclear
*VcDof25*	VaccDscaff19-processed-gene-236.11	VaccDscaff19:23614873-23616681	789	262	9.30	28,419.66	Nuclear
*VcDof26*	VaccDscaff19-snap-gene-338.49	VaccDscaff19:33815798-33819654	1182	393	6.00	43,431.08	Nuclear
*VcDof27*	VaccDscaff19-augustus-gene-349.24	VaccDscaff19:34887825-34889658	1239	412	9.93	45,154.72	Nuclear
*VcDof28*	VaccDscaff20-augustus-gene-371.28	VaccDscaff20:37159924-37162529	990	329	8.13	35,787.13	Nuclear
*VcDof29*	VaccDscaff21-processed-gene-152.13	VaccDscaff21:15189887-15192636	1014	337	8.91	36,226.51	Nuclear
*VcDof30*	VaccDscaff21-augustus-gene-306.24	VaccDscaff21:30639419-30644218	1245	414	6.47	45,615.07	Nuclear
*VcDof31*	VaccDscaff22-augustus-gene-267.28	VaccDscaff22:26762373-26763954	753	250	8.34	27,207.32	Nuclear
*VcDof32*	VaccDscaff22-processed-gene-275.2	VaccDscaff22:27553163-27555116	939	312	8.81	33,950.99	Nuclear/ Peroxisome
*VcDof33*	VaccDscaff24-snap-gene-94.38	VaccDscaff24:9471913-9475742	1197	398	5.76	44,173.03	Nuclear
*VcDof34*	VaccDscaff24-processed-gene-229.1	VaccDscaff24:22899664-22900548	885	294	8.13	32,341.95	Nuclear
*VcDof35*	VaccDscaff24-processed-gene-290.7	VaccDscaff24:29059975-29060412	438	145	10.48	16,520.86	Nuclear
*VcDof36*	VaccDscaff27-snap-gene-20.34	VaccDscaff27:2024971-2025485	357	118	10.56	13,621.80	Nuclear
*VcDof37*	VaccDscaff27-augustus-gene-35.18	VaccDscaff27:3516664-3518800	1026	341	9.14	35,675.48	Nuclear/ Peroxisome
*VcDof38*	VaccDscaff28-augustus-gene-4.38	VaccDscaff28:457494-460214	993	330	8.13	35,901.23	Nuclear
*VcDof39*	VaccDscaff29-snap-gene-153.37	VaccDscaff29:15276983-15278775	960	319	9.16	34,455.72	Nuclear
*VcDof40*	VaccDscaff30-augustus-gene-326.23	VaccDscaff30:32673932-32678040	1482	493	5.83	53,701.66	Nuclear/ mitochondrial
*VcDof41*	VaccDscaff31-processed-gene-100.12	VaccDscaff31:10070463-10071110	648	215	9.05	22,434.25	Nuclear
*VcDof42*	VaccDscaff33-augustus-gene-185.34	VaccDscaff33:18572264-18573956	966	321	9.14	34,766.97	Nuclear
*VcDof43*	VaccDscaff34-augustus-gene-312.13	VaccDscaff34:31205413-31207358	969	322	9.20	33,725.28	Nuclear/ Peroxisome
*VcDof44*	VaccDscaff35-processed-gene-123.5	VaccDscaff35:12329415-12329894	480	159	9.27	17,650.15	Nuclear
*VcDof45*	VaccDscaff35-augustus-gene-253.36	VaccDscaff35:25292011-25293987	879	292	8.10	31,873.07	Nuclear
*VcDof46*	VaccDscaff37-augustus-gene-143.22	VaccDscaff37:14293905-14295828	837	278	9.40	30,154.54	Nuclear
*VcDof47*	VaccDscaff40-processed-gene-12.4	VaccDscaff40:1275799-1276467	669	222	6.64	23,658.98	Nuclear
*VcDof48*	VaccDscaff41-processed-gene-58.8	VaccDscaff41:5852050-5852715	666	221	6.64	23,614.92	Nuclear
*VcDof49*	VaccDscaff46-augustus-gene-100.28	VaccDscaff46:10034713-10036487	747	248	8.34	27,093.22	Nuclear
*VcDof50*	VaccDscaff46-snap-gene-129.47	VaccDscaff46:12897112-12899008	936	311	8.58	33,975.09	Nuclear/ Peroxisome
*VcDof51*	VaccDscaff48-augustus-gene-102.22	VaccDscaff48:10277144-10279070	864	287	8.81	31,974.36	Nuclear

### Phylogenetic tree of the Dof transcription factors in blueberry

**Figure 1:**
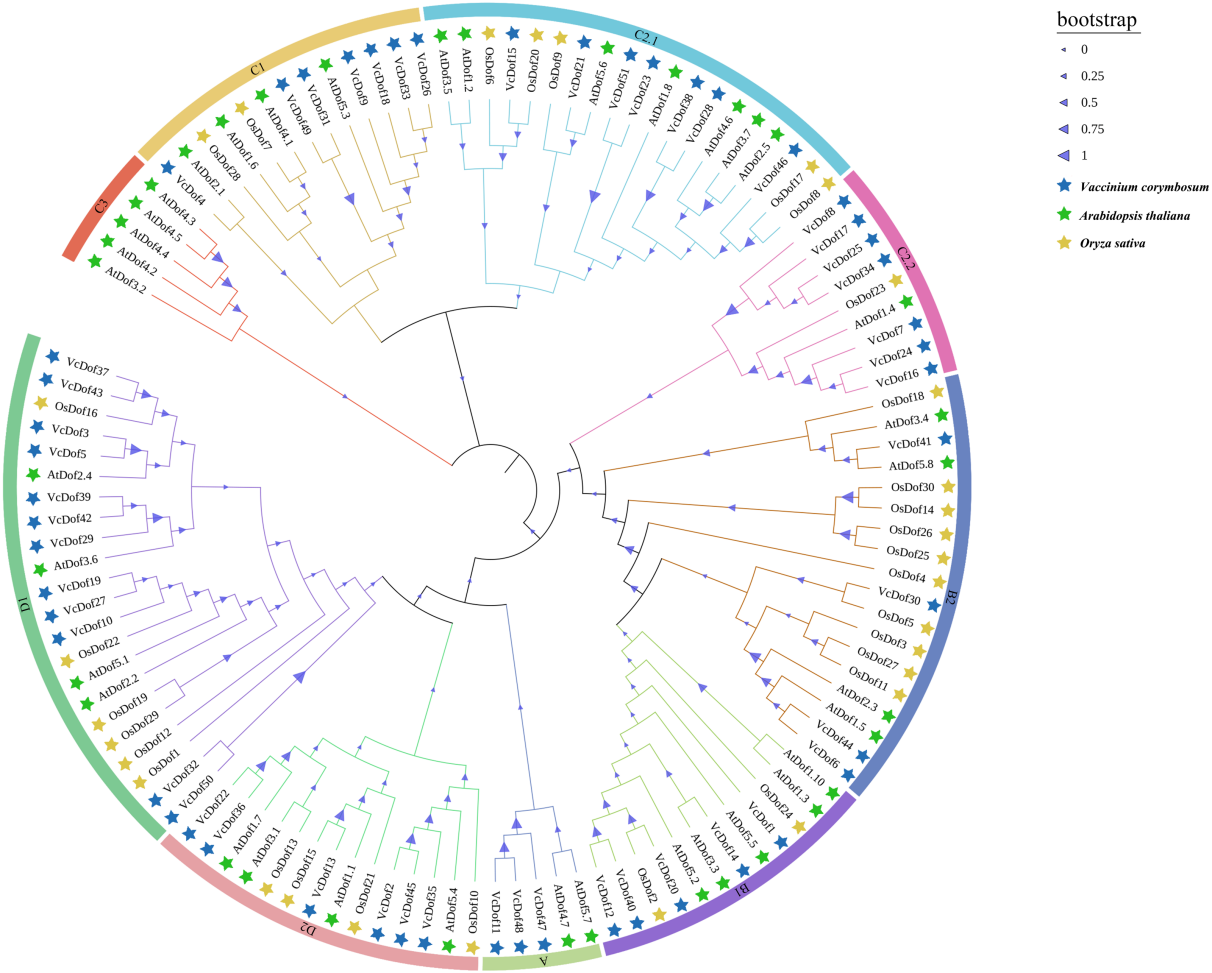
Phylogenetic tree of the Arabidopsis, rice and blueberry Dof transcription factors. The nine subfamilies were shown in different colors. The blue filled pentagram denoted *VcDof* genes; the green-filled pentagram denoted *AtDof* genes; the yellow-filled pentagram denoted *OsDof* genes.

To further understand the evolutionary relationship of Dof TFs, the amino acid sequences of Dof genes in blueberry, Arabidopsis, and rice was compared using the MegaX software. The results showed that (Fig. 1), all Dof genes were divided into four main subfamilies (subfamilies A–D), which could be divided into multiple subfamilies, A, B1, B2, C1, C2.1, C2.2, C3, D1, and D2 with supported bootstrap values. Analysis of each subfamily found that the D1 subfamily contained the largest number of Dof TFs, consistent with Wei’s finding that the D1 subfamily had the most members in the eggplant phylogenetic tree (Wei et al., 2018). The Dof TFs of dicotyledonous blueberry, Arabidopsis, and monocotyledonous rice did not show apparent separation in the phylogenetic tree, indicating that in the long evolutionary process, Dof TFs did not appear obvious differentiation between monocotyledonous and dicotyledonous plants, may contain similar functions. Therefore, the Dof TFs are relatively conserved in the evolution of plants. Interestingly, *VcDof* and *OsDof* genes did not appear in the C3 subfamily.

### Gene structure and conserved motifs analysis of the Dof transcription factors

**Figure 2:**
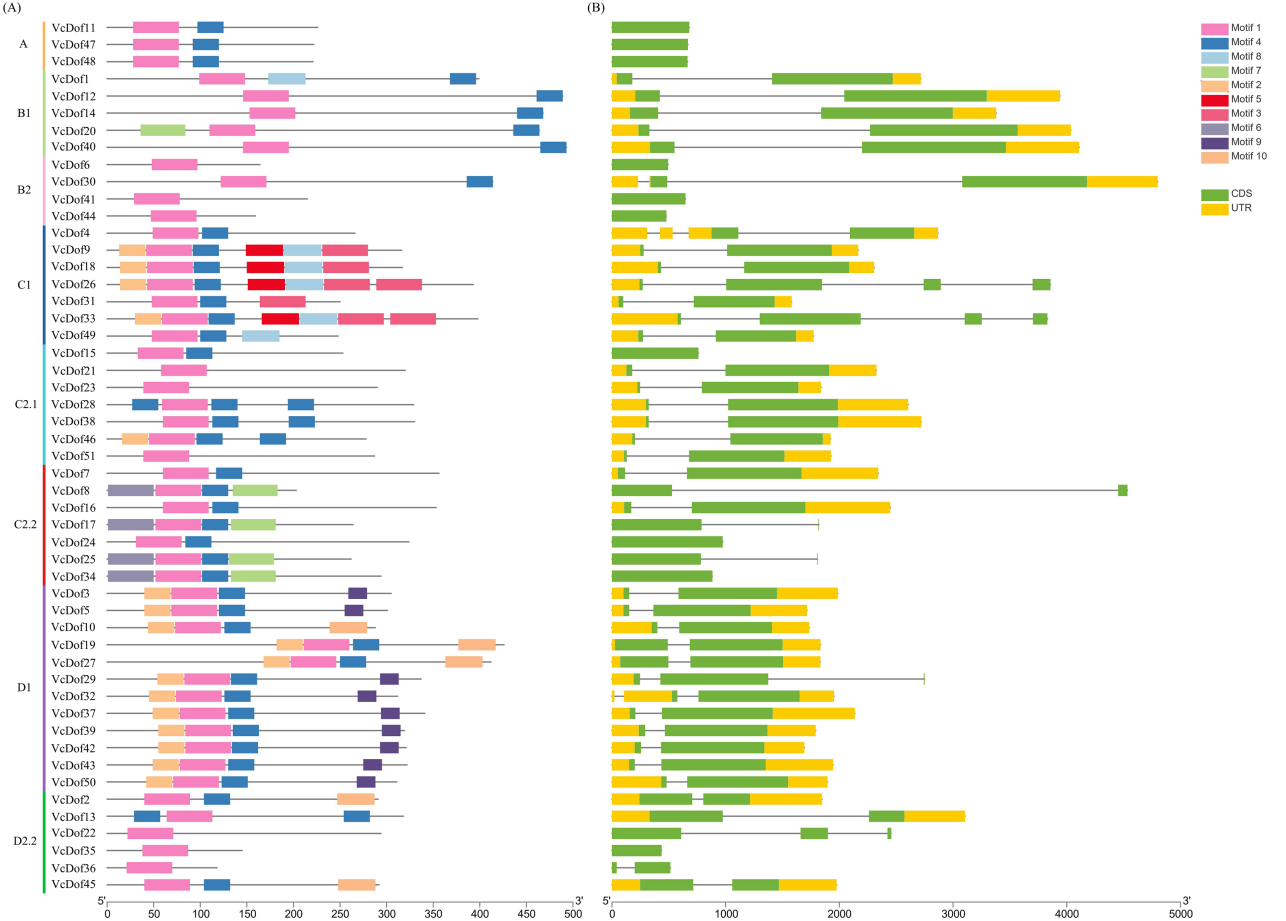
Conserved protein motifs and gene structure analysis of Dof transcription factors in blueberry. (A) Identified conserved protein motifs in the *VcDof* genes. Each motif was indicated with a specific color, different colors of lines denoted the different subfamilies; (B) Gene structure of the *VcDof* genes. The green box represented the CDS region and yellow box represented the UTR, and the grey lines represented introns.

In order to clarify the genetic structure of the *VcDof* genes, the conserved motifs of *VcDof* genes were analyzed by MEME. The results showed that ten motifs (motif1-motif10) were obtained (Fig. S1). Motif1 was the conserved motif of the Dof transcription factor, and motif1 was contained in each identified *VcDof* gene, proving the identification results reliability. The arrangement and number of motifs in each subfamily were the same (Fig. 2A). Studies have shown that the gene’s distribution of exons and introns is closely related to the evolution of the gene family. Gene structure analysis results show that *VcDof* genes contain coding sequence (CDS) and untranslated region (UTR) with different numbers and lengths (Fig. 2B). The number of introns in *VcDof* genes was 0–3, 66.7% of *VcDof* genes contained only one intron, *VcDof4, VcDof26,* and *VcDof33* had three introns, and members of the same subfamily had similar numbers of introns. There are differences in intron length between different subfamilies, possibly due to the absence or increase of introns in the long-term evolution process. *VcDof* genes belong to the exon-poor subgroup, indicating that blueberry Dof TFs are relatively conservative in the evolutionary process (Hu et al., 2015a; Hu et al., 2015b).

### Collinearity gene pair and divergence time analysis of the Dof transcription factors in blueberry

**Figure 3:**
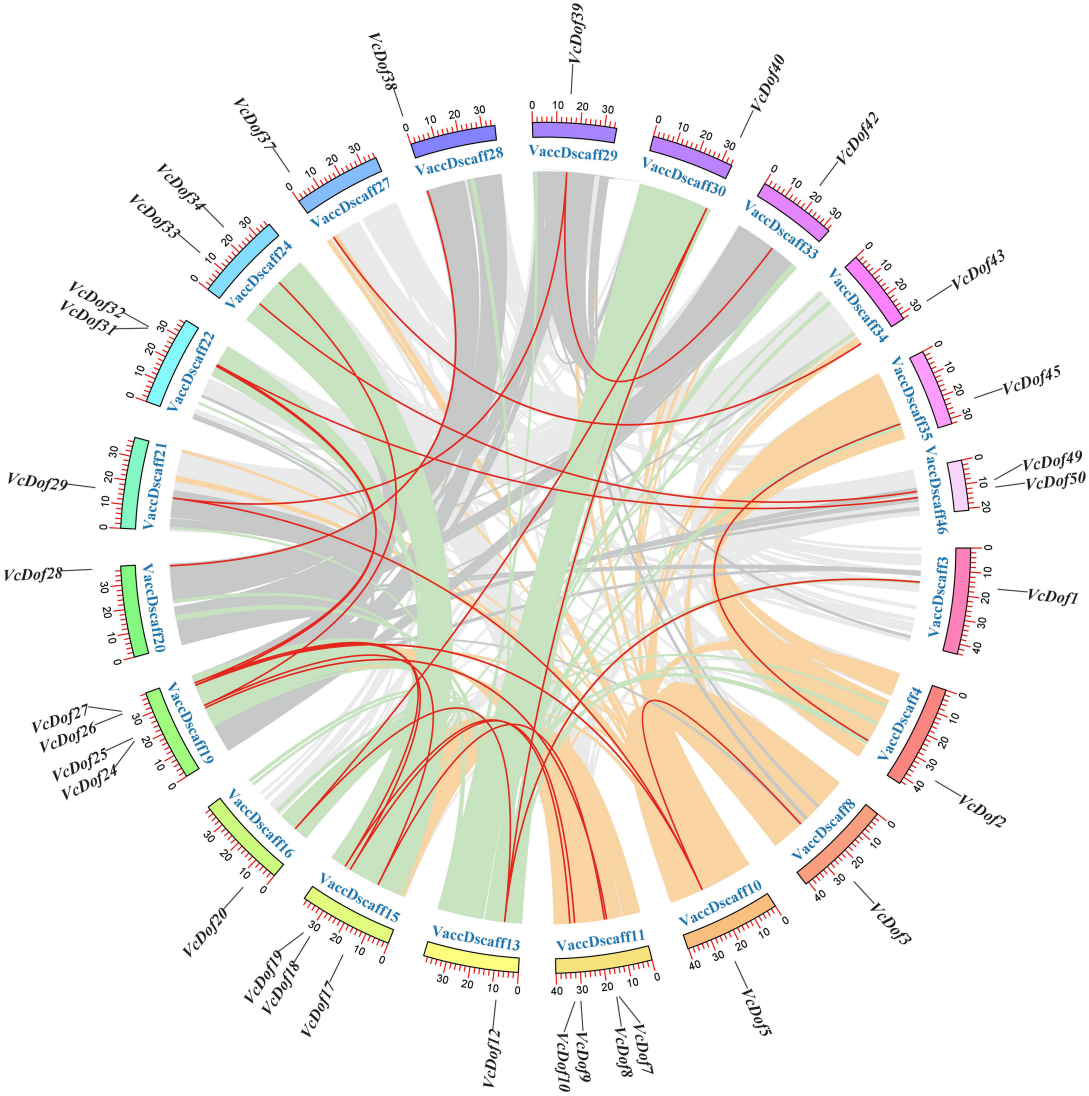
Chromosomal location and collinearity analysis of Dof TFs in blueberry. The outer color ring box in the circle represented the chromosome scaffold, the color part inside the circle was the blueberry genome collinear region, and the red line was the blueberry Dof TFs collinear gene pair.

Due to the incomplete splicing and chromosomal assembly of the blueberry genome, the genes can only be located on the chromosomal scaffold of the blueberry. The results showed that (Fig. 3), 24 pairs of collinearity genes were found on 21 chromosome scaffolds, showing uneven distribution; VaccDscaff19 and VaccDscaff11 contained the most collinearity genes with four pairs. We have found only a pair of collinearity genes in the chromosome 9 scaffold. The non-synonymous/synonymous mutation ratio (Ka/Ks) is a common tool to study the selection pressure of gene evolution. This study calculated the evolutionary selection pressure of *VcDof* collinearity gene pairs. The results showed that the Ka/Ks ratio of 91.67% collinear gene pairs were all less than 1, subjected to purifying selection during the evolution process (Table 2), while *VcDof7-VcDof24* and *VcDof32-VcDof50* were subjected to positive selection. Overall, the *VcDof* genes were subjected to intense purifying selection pressure during the evolution process. Whole-genome duplication (WGD) events provided the main driving force for the evolution of the *VcDof* genes. The estimated time of divergent events between collinear genes was 0.2699–95.6425 Mya ago.

**Table 2:** The evolution selection pressure and divergence time of Dof TFs in blueberry.

Duplicated pair	Duplicated model	Ka	Ks	Ka/Ks	Selection pressure	Divergence time (Mya)
*VcDof1-VcDof12*	WGD	0.365234001	2.486705939	0.146874625	purify selection	95.6425361
*VcDof2-VcDof45*	WGD	0.001505269	0.060032031	0.02507443	purify selection	2.30892426
*VcDof3-VcDof5*	WGD	0.002911212	0.033338822	0.087321976	purify selection	1.282262382
*VcDof5-VcDof27*	WGD	0.359266416	1.723444937	0.208458308	purify selection	66.28634373
*VcDof5-VcDof29*	WGD	0.417288029	1.517602966	0.274965217	purify selection	58.36934483
*VcDof7-VcDof24*	WGD	0.013538274	0.00899561	1.504986721	positive selection	0.345985003
*VcDof8-VcDof17*	WGD	0.063654913	0.144930931	0.439208611	purify selection	5.574266561
*VcDof9-VcDof18*	WGD	0.001353791	0.009646435	0.140341064	purify selection	0.37101674
*VcDof10-VcDof19*	WGD	0.004531736	0.025426164	0.178231206	purify selection	0.977929375
*VcDof12-VcDof20*	WGD	0.149983742	0.709859291	0.21128658	purify selection	27.30228042
*VcDof12-VcDof40*	WGD	0.007081265	0.040226191	0.176036186	purify selection	1.547161197
*VcDof17-VcDof25*	WGD	0.005002798	0.027627433	0.181080807	purify selection	1.062593578
*VcDof18-VcDof26*	WGD	0.032607364	0.036268486	0.899055024	purify selection	1.394941757
*VcDof19-VcDof27*	WGD	0.003165675	0.007017595	0.451105363	purify selection	0.269907502
*VcDof20-VcDof40*	WGD	0.14460691	0.708639164	0.204062825	purify selection	27.25535246
*VcDof25-VcDof34*	WGD	0.006670416	0.010969117	0.608108789	purify selection	0.421889112
*VcDof26-VcDof31*	WGD	0.224480814	0.565207401	0.397165382	purify selection	21.7387462
*VcDof27-VcDof32*	WGD	0.293672643	0.906127367	0.324096428	purify selection	34.85105259
*VcDof28-VcDof38*	WGD	0.001305483	0.018377642	0.071036498	purify selection	0.706832396
*VcDof29-VcDof39*	WGD	0.018104628	0.036550818	0.495327557	purify selection	1.405800703
*VcDof32-VcDof50*	WGD	0.025707622	0.009371461	2.743181808	positive selection	0.360440817
*VcDof33-VcDof49*	WGD	0.220819905	0.542497777	0.407042967	purify selection	20.86529913
*VcDof37-VcDof43*	WGD	0.008295015	0.038690527	0.214393966	purify selection	1.488097177
*VcDof39-VcDof42*	WGD	0.015991336	0.034226471	0.467221286	purify selection	1.316402732

### Promoters analysis of the Dof transcription factors in blueberry

**Figure 4:**
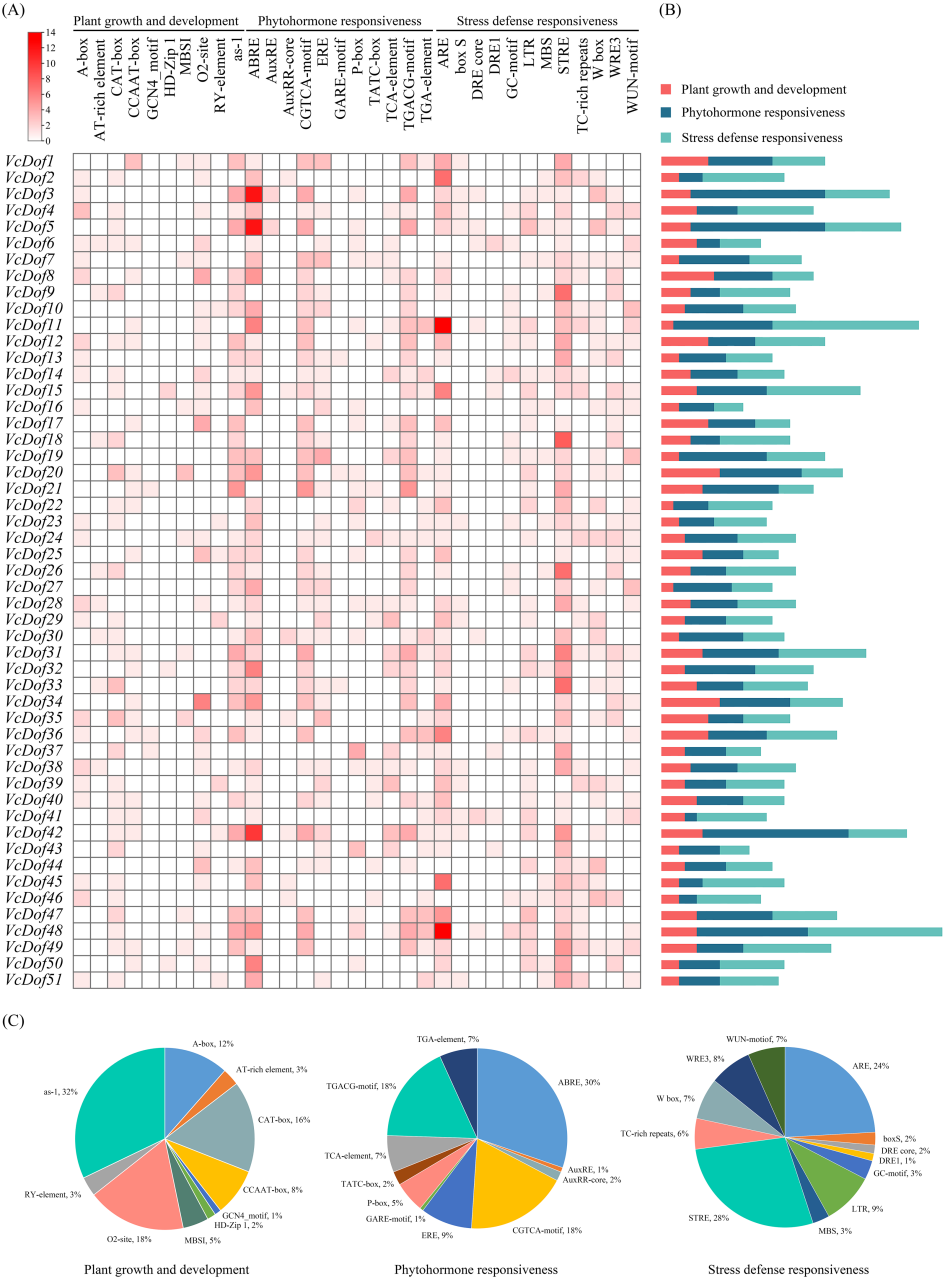
The cis-acting elements of Dof TFs in blueberry. (A) The gradient red colors indicate the number of cis-acting elements; (B) color-coded histograms indicate the number of cis-acting elements of genes in each category; (C) pie charts show the proportion of different cis-acting elements in each category.

Transcription factors bind to cis-acting elements of promoters to initiate gene transcription, and promoters are key factors that determine the spatiotemporal expression and transcription levels of genes (Brázda, Bartas & Bowater, 2021). In this study, PlantCARE was used to analyze the cis-acting promoter elements of 2,000 bp upstream of the start site of the *VcDof* genes (Fig. 4). All cis-acting promoters were classified into three categories according to their functions: plant growth and development, phytohormone responsiveness, and stress defense responsiveness. Plant growth and development-related elements analysis showed that the ‘as-1 promoter’ was most distributed in *VcDof* genes, accounting for 32%, and 18% with the ‘O2-site’ promoter related to cis-acting regulatory element involved in zein metabolism regulation. Among the phytohormone responsiveness elements, ABRE (abscisic acid responsiveness) was the most numerous promoter (30%), which was the ABA-responsive element. 86.3% of the *VcDof* genes promoter contained ABRE elements, suggesting that the *VcDof* genes played an important role in regulating abscisic acid. STRE (thermal stress responsive element) was the promoter with the highest proportion (28%) in the stress defense responsiveness elements, and it also contained TC-rich repeats (6%), LTR (long terminal repeats, 9%), and MBS (MYB binding site, 3%) promoters. Studies have shown that these promoters are involved in defense and stress tolerance (Wang et al., 2018). *VcDof11* gene contains 25 abiotic stress-related promoters, one of the most stress tolerance elements in *VcDof* genes. It is speculated that *VcDof11* plays an important role in the response of blueberry to abiotic stress.

### Expression profiles of blueberry Dof transcription factors in different tissues and fruit development stages

**Figure 5:**
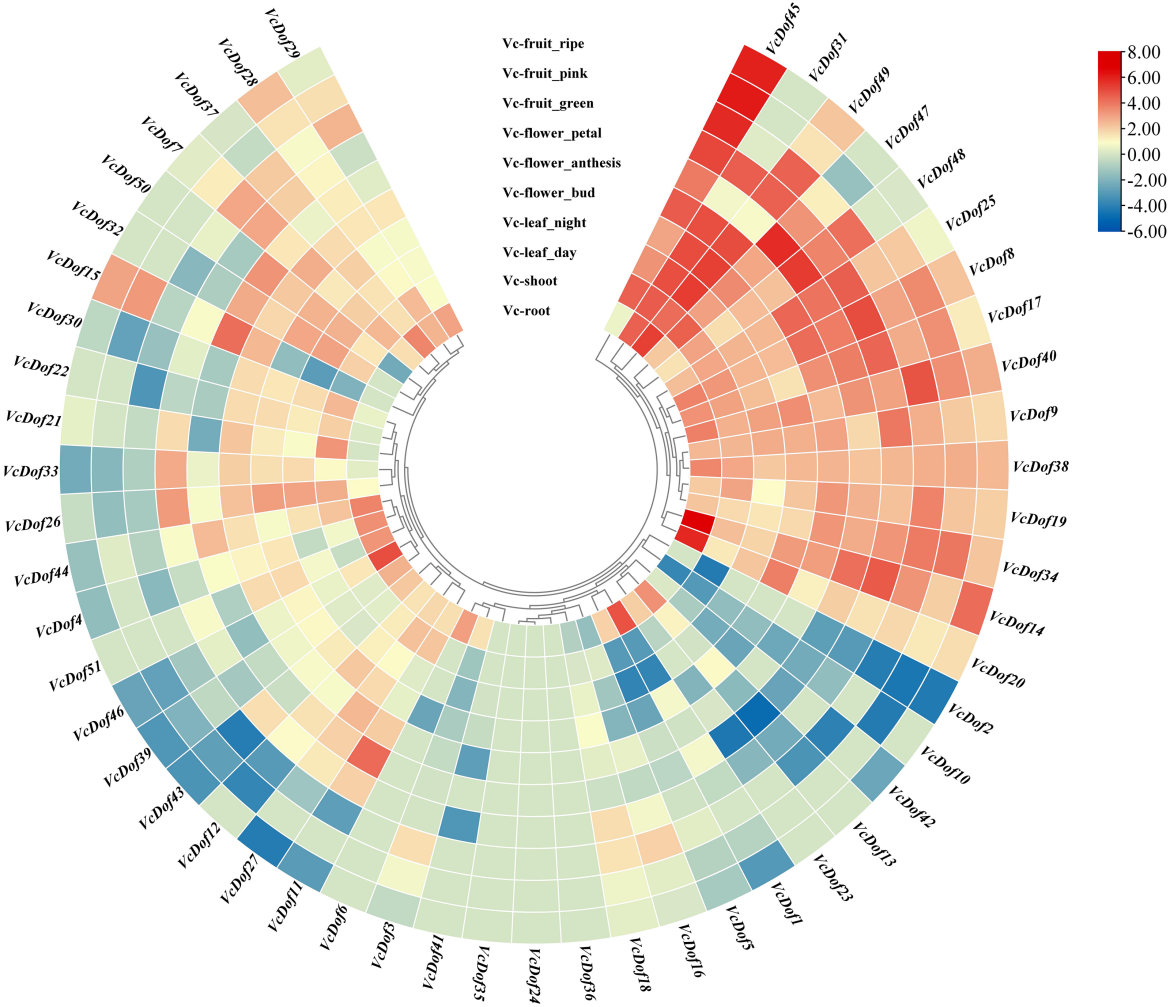
Expression profiles of Dof TFs in different tissues and fruit development stages of blueberry. The blue or red indicated lower or higher expression levels of each transcript in each sample, respectively.

Using the RNA-seq data, we have explored the expression profile of different tissues at different time points. A heatmap of *VcDof* genes for root, shoot, leaf (day and night), flower (bud, anthesis, and petal), and fruit (green, pink, and ripe) is available (Fig. 5). It was found that, except for *VcDof24*, *VcDof35*, and *VcDof36*, other blueberry Dof TFs were detected in various blueberry tissues. Overall, all blueberry Dof TFs were highly expressed in the root. Among them, a total of 15 *VcDof* genes, *VcDof8, VcDof9, VcDof14, VcDof17, VcDof19, VcDof20, VcDof25, VcDof31, VcDof34, VcDof38, VcDof40, VcDof45, VcDof47, VcDof48*, and *VcDof49* were highly expressed in different tissues and fruit development stages of blueberry, suggesting that they may play an important positive regulatory role in blueberry growth and development. However, the expression levels of 7 *VcDof* genes, including *VcDof1, VcDof2, VcDof5, VcDof10, VcDof13, VcDof23*, and *VcDof42* were relatively low in different tissues and fruit development stages of blueberry. Therefore, they may play a negative feedback regulation role in blueberry growth and fruit ripening. Interestingly, the expression level of *VcDof45* gradually increased during the process of blueberry fruit ripening (green to ripe), while the expression level of *VcDof2* gradually decreased. It is speculated that these two genes play important regulatory roles in the development of blueberry fruit. The remaining blueberry Dof TFs showed different expression levels in different tissues, which may have different biological functions.

### Expression profiles of blueberry Dof transcription factors in response to salt, drought and abscisic acid

Previous studies have shown that Dof TFs are widely involved in the biological processes of plants responding to abiotic stresses. To clarify the possible biological functions of blueberry Dof TFs under abiotic stress, we selected eight genes with stress defense response elements in promoters. We performed qRT-PCR to analyze their expression profiles under the abiotic stress.

**Figure 6:**
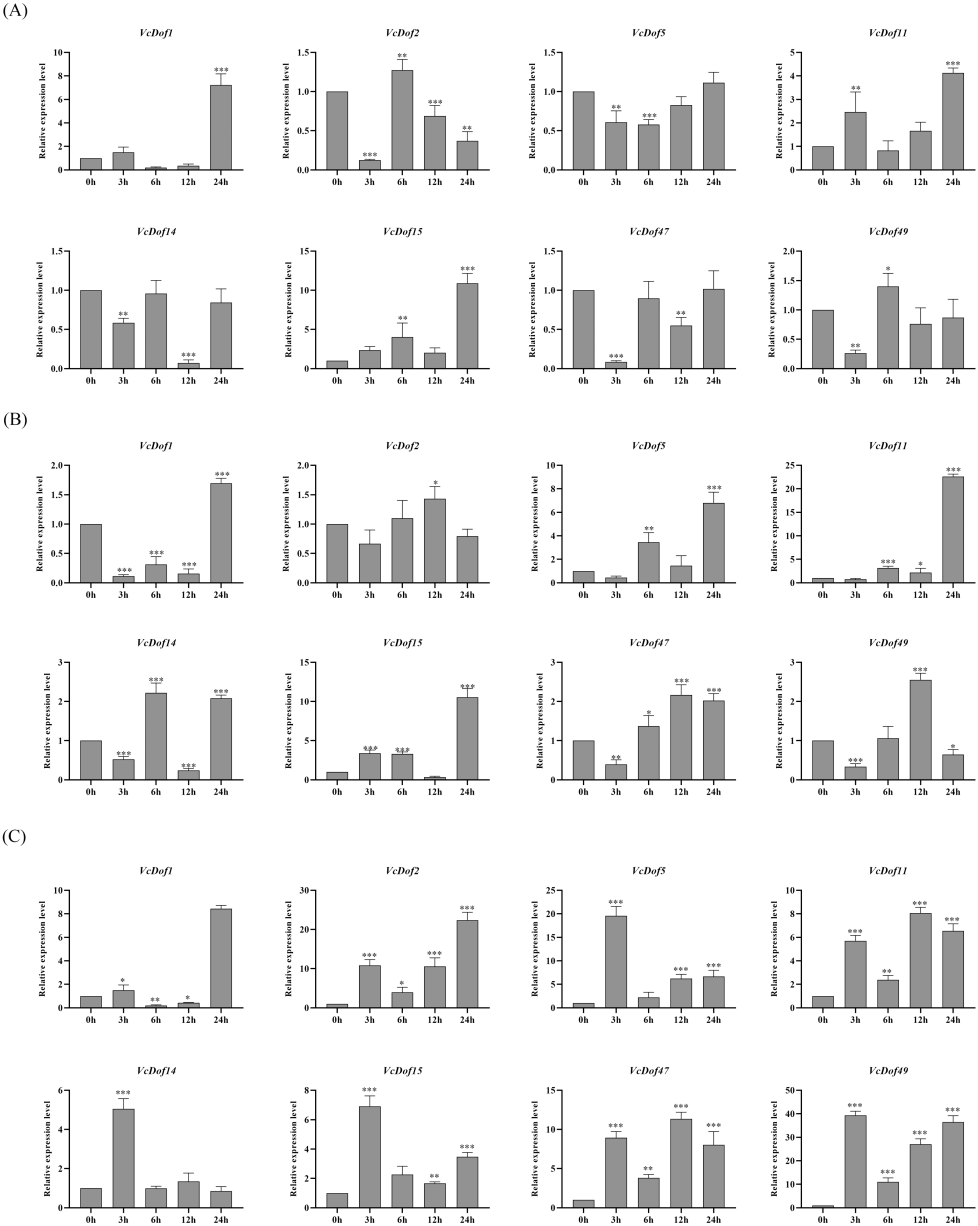
Expression profiles of blueberry Dof TFs in response to salt, drought and abscisic acid. (A) Expression profiles of *VcDof* genes under salt stress. (B) Expression profiles of *VcDof* genes under drought stress. (C) Expression profiles of *VcDof* genes under abscisic acid. Error bars indicate standard deviation, and asterisks indicate significant differences between the control and treatments, ^∗^*p* < 0.05, ^∗∗^*p* < 0.01, ^∗∗∗^*p* < 0.001.

The results showed that the expression of *VcDof* genes was regulated early by salt stress (Fig. 6A). With the prolongation of stress time, *VcDof1, VcDof11*, and *VcDof15* showed an up-regulated expression trend, and their relative expression levels were the highest at 24 h, 8.54, 3.26, and 9.07 times that of the control group, significant differences have been noticed. *VcDof2* showed a down-regulated expression trend, and the relative expression level was the lowest at 24 h, which was significantly lower than the control group. Short-term drought stress also caused changes in the relative expression levels of *VcDof* genes (Fig. 6B). The relative expression levels of *VcDof5, VcDof11*, and *VcDof15* at 24 h of drought stress were 7.35, 18.47, and 14.48 times higher than those in the control group. The expression trend was down-regulated at 0–12 h of stress and up-regulated at 24 h. In general, the eight blueberry Dof TFs responded positively to drought stress in the early stage and were mainly up-regulated. Under ABA stress (Fig. 6C), the relative expression levels of *VcDof* genes changed significantly, and *VcDof1* and *VcDof2* were the highest at 24 h stress, which was 9.59 and 25.28 times that of the control group. The relative expression levels of *VcDof5, VcDof14, VcDof15*, and *VcDof49* were the highest at 3 h of stress, which were 20.40, 4.30, 5.13, and 29.26 times that of the control group. Blueberry Dof TFs responded positively to ABA stress, and the relative expression changed significantly.

## Discussion

### Identification and characterization of *VcDof* genes

As a class of transcription factors with C2C2-Dof zinc finger structure in plants, Dof plays an important role in plant growth and development and stress resistance (Venkatesh & Park, 2015). In this study, we have identified 51 *VcDof* genes in blueberry, which was lower than the number of Dof TFs in Chinese cabbage (76 *BraDof* genes, Ma et al., 2015). The number of Dof TFs was similar to that contained in maize (46 *ZnDof* genes, Chen & Cao, 2015), higher than that of Dof TFs in Arabidopsis (36 *AtDof* genes, Liu et al., 2020), tomato (34 *SlDof* genes, Cai et al., 2013), and pepper (33 *CaDof* genes, Wu et al., 2016). The results of multiple sequence alignment (Fig. S2) showed that all blueberry Dof TFs contained zinc-finger Dof conserved domains, and the results of gene structure and motif analysis showed that the *VcDof* genes in the same subfamily had similar exon or intron structure and motif ordering prove that blueberry Dof TFs are highly conserved (Wang et al., 2021a; Wang et al., 2021b).

### Collinearity and duplication events analysis of *VcDof* genes

The Dof TFs of Arabidopsis, rice, and blueberry were constructed using MegaX to construct a phylogenetic tree, and the results showed that all Dof TFs were divided into four families (A–D) and nine subfamilies (A, B1, B2, C1, C2. 1. C2.2, C3, D1, and D2), in which the blueberry Dof gene was not found in the C3 subfamily. This is consistent with the results of studies by Wen and Lijavetzky found that cucumber *CsDofs* and rice *OsDofs* were lost in the C3 subfamily (Lijavetzky, Carbonero & Vicente-Carbajosa, 2003; Wen et al., 2016).

Gene duplication often occurs among gene family members, making gene function-specific and diverse, which is one of the main driving forces for plant genome evolution. Whole-genome duplication (WGD) promotes chromosomal recombination, gene doubling, and diversification of gene functions (Van de Peer, Maere & Meyer, 2009). Bowers’s study showed two recent WGD events occurred in Arabidopsis: the *β* whole-genome duplication event and the *α* whole-genome duplication event (Bowers et al., 2003). The amplification sources of 36 Arabidopsis Dof TFs are mainly *β*-genome duplication events and tandem duplication events (Wang, Tan & Paterson, 2013). The results of collinearity analysis showed that there were 24 pairs of collinearity gene pairs in 51 blueberry Dof TFs, all of which belonged to WGD. The Ka/Ks ratio of genes except *VcDof7-VcDof24* and *VcDof32-VcDof50* were less than 1, indicating that the WGD event of *VcDof* genes was the result of purifying selection. The repeated divergence events of monocotyledonous and dicotyledonous plants occurred before 170-235 Mya (Blanc & Wolfe, 2004). In this study, the divergence time of the collinearity gene of blueberry Dof TFs was before 0.2699–95.6425 Mya and later than that of monocotyledonous plants and dicotyledonous plants, which also explained why the Dof TFs of dicotyledonous plants Arabidopsis, blueberry and monocotyledonous plants rice in the evolutionary tree of this study did not have obvious segregation in clustering.

### Tissue specific expression of *VcDof* genes

The analysis of gene expression patterns can reflect the function of genes to a certain extent. The tissue expression analysis results of this study showed that blueberry Dof TFs were differentially expressed in different tissues and developmental stages, but the overall expression was higher in the root. This is consistent with the experimental results that the Dof transcription factors were highly expressed in cucumber and pepper root tissues (Wu et al., 2016; Wen et al., 2016). The expression levels of *VcDof2* and *VcDof45* continued to change during blueberry flowering and fruit development. Previous studies have shown that *AtDof4.1*, as a transcription inhibitor, delays the flowering of Arabidopsis and inhibits the development of reproductive organs, resulting in smaller leaves, flowers, and siliques (Ahmad et al., 2013). In rice, under dark conditions, the *OsDof12* (*Rdd1*) gene was inhibited, while the expression was up-regulated under light conditions. Over-expression of *OsDof12* (*Rdd1*) significantly delayed the flowering time of transgenic rice under long-day conditions, and the downstream genes *Hd3a* and *OsMADS14* were up-regulated. After interfering with *OsDof12* (*Rdd1*) gene expression, the flowering time of rice was delayed, and rice’s grain size and thousand-grain weight were significantly reduced (Li et al., 2009). It is speculated that *VcDof45* and *VcDof2* play important roles in flowering regulation and fruit development of blueberries.

### Potential role of *VcDof* genes in response to abiotic stress

Dof TFs play an important role in abiotic stress in plants. This study selected eight *VcDof* genes for expression pattern analysis under abiotic stress. The results showed that the relative expression levels of *VcDof1, VcDof11*, and *VcDof15* under salt, drought, and ABA short-term induction stress were significantly higher than those in the control group, showing an upward expression trend. The *VcDof* gene expression differed among the three stresses, but they could respond positively to stress. According to previous studies, *StDof4, StDof5*, and *StDof11b* were up-regulated under salt and drought stress in potatoes and positively responded (Venkatesh & Park, 2015). Most *BraDof* genes were rapidly up-regulated under salt, drought, heat, and cold stress in Chinese cabbage (Ma et al., 2015). *TaDof2, TaDof3*, and *TaDof6* were up-regulated, and soluble protein synthesis increased under drought stress in wheat (Liu et al., 2020). Therefore, we speculate that *VcDof1*, *VcDof11* and *VcDof15* are positive regulators in the process of blueberry resisting abiotic stress and assume important functions, but their specific regulatory mechanisms still need further study.

## Conclusions

In general, 51 conserved *VcDof* genes were identified in the present study, distributed in eight subfamilies. Colinearity and evolution analysis showed that the main driving force of gene duplication of the *VcDof* was WGD, which was purify selected in the evolution process. The gene divergence event occurred after the divergence between monocotyledonous and dicotyledonous plants. The results of tissue expression analysis showed that *VcDof2* and *VcDof45* might play important roles in blueberry flowering and fruit development. *VcDof1, VcDof11*, and *VcDof15* can respond positively and up-regulate expression under abiotic stress, which may play an important role in blueberry defense against abiotic stress.

##  Supplemental Information

10.7717/peerj.14087/supp-1 Supplemental Information 1 Primer sequences used in the experiment

10.7717/peerj.14087/supp-2 Supplemental Information 2 Raw data for qRT-PCR

10.7717/peerj.14087/supp-3 Supplemental Information 3 The sequence used in the present research

10.7717/peerj.14087/supp-4 Supplemental Information 4 Motifs of Dof proteins in blueberry

10.7717/peerj.14087/supp-5 Supplemental Information 5 Multiple sequence alignment of Zinc-finger domain (marked with top) of blueberry Dof TFs proteins

## Additional Information and Declarations

### Data Availability

The following information was supplied regarding data availability: The raw data is available in the Supplementary File.
