# Supplementary figures and images for: Genome-wide identification, phylogenetic and expression pattern analysis of Dof transcription factors in blueberry (Vaccinium corymbosum L.)
Source: PeerJ. 2022 Oct 3;10:e14087. doi: 10.7717/peerj.14087 (PMC9536302; doi:10.7717/peerj.14087)

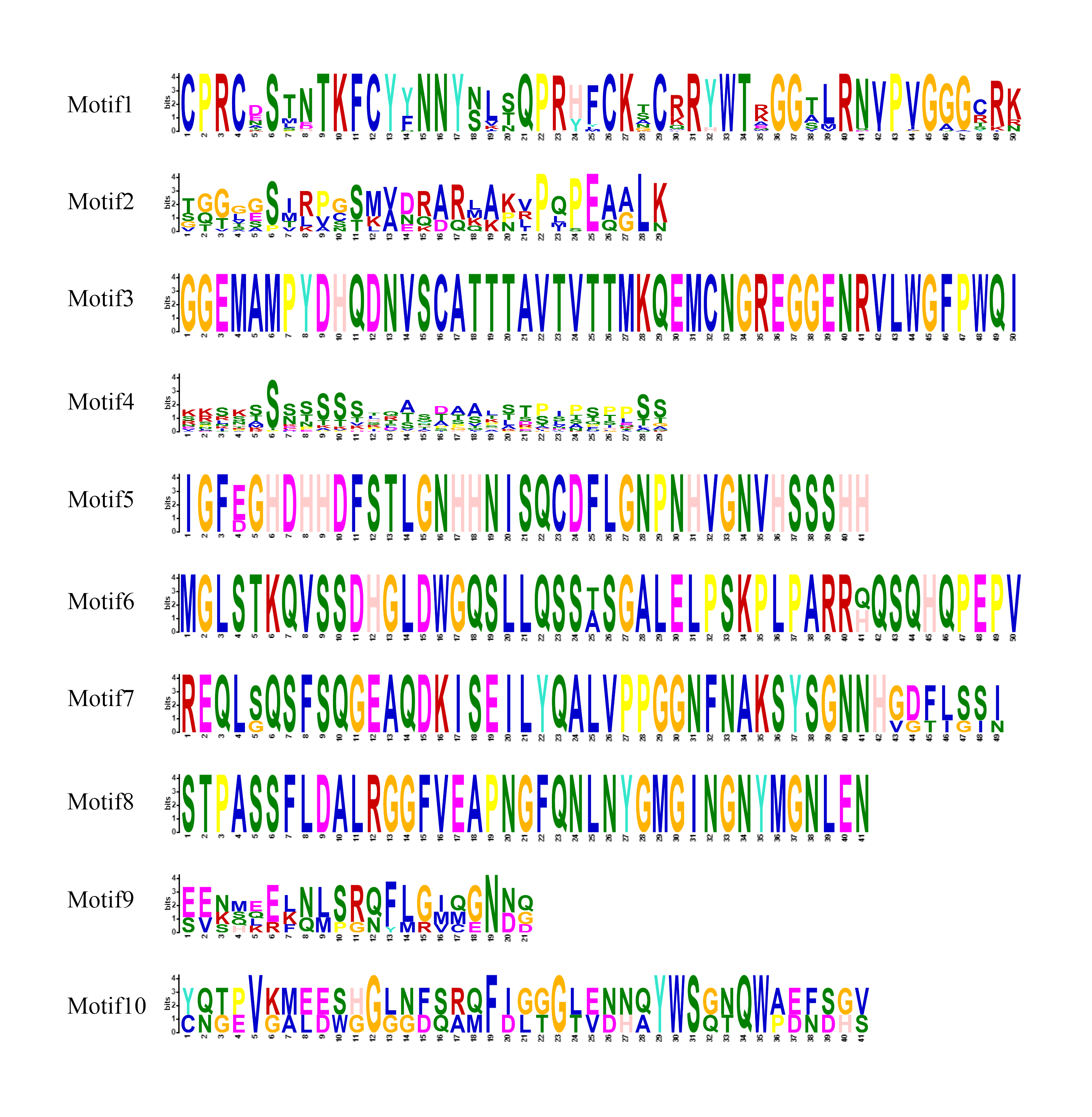

Supplement: Supplemental Information 4 [file peerj-10-14087-s004.png]

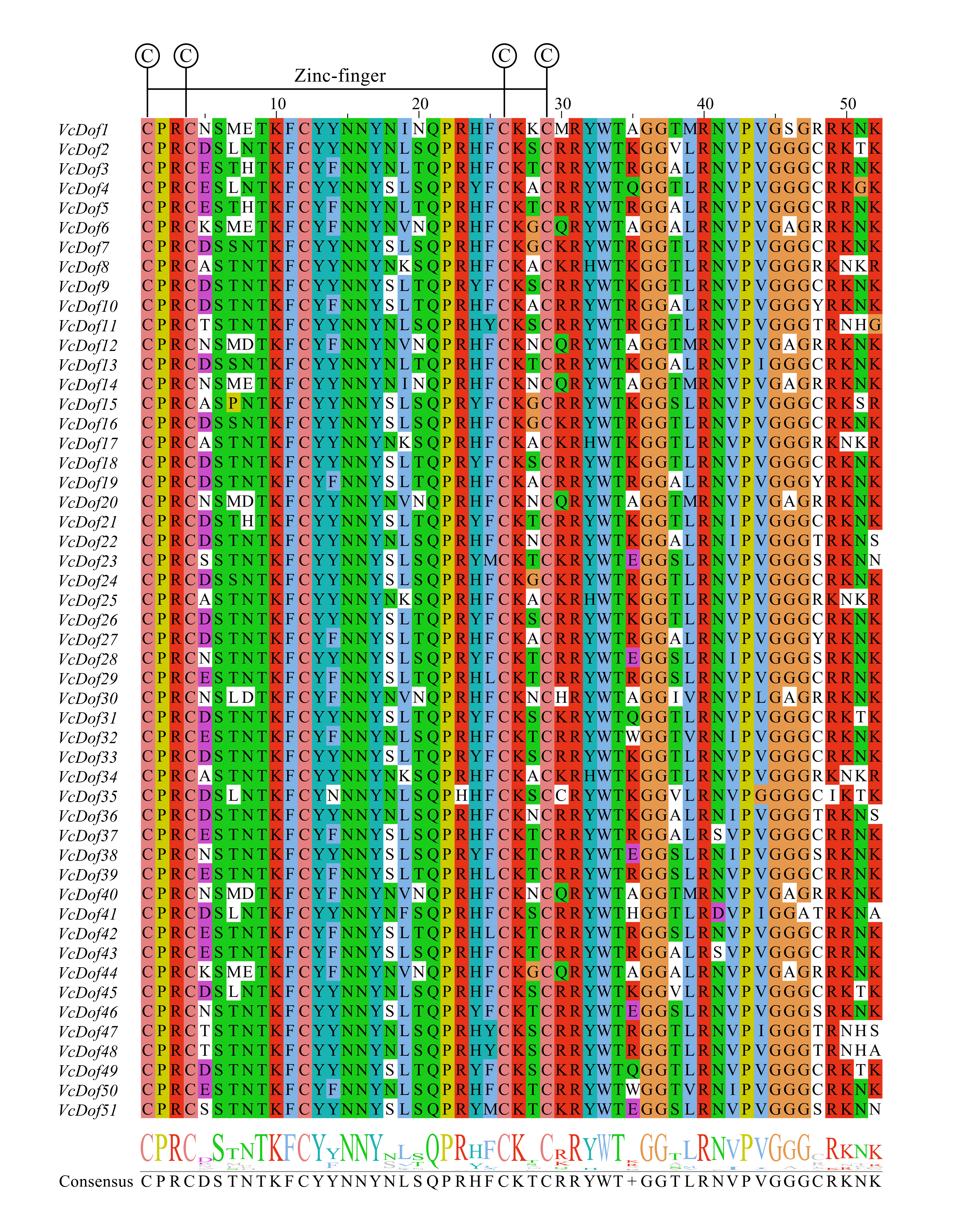

Supplement: Supplemental Information 5 [file peerj-10-14087-s005.png]
